# Neuroimmunology: An Expanding Frontier in Autoimmunity

**DOI:** 10.3389/fimmu.2015.00206

**Published:** 2015-04-29

**Authors:** Romana Höftberger

**Affiliations:** ^1^Institute of Neurology, Medical University of Vienna, Vienna, Austria

**Keywords:** autoimmune encephalitis, anti-NMDAR encephalitis, limbic encephalitis, anti-neuronal antibodies, tissue-based assay

## Abstract

Anti-neuronal autoimmune encephalitis (AIE) comprises a recently characterized group of immune-mediated disorders that result in limbic, multifocal, or diffuse encephalitis due to direct interaction of autoantibodies with neuronal surface or synaptic proteins. The pathological effects of the autoantibodies vary according to the target antigen but when they are removed, neuronal dysfunction is commonly reversed. Ongoing research on AIE constantly increases the number of novel autoantibodies and expands the spectrum of neurological syndromes that are important in the differential diagnosis of psychiatric illness, dementia, or viral encephalitis. This review summarizes recent advances in AIE, focusing on pathogenetic mechanisms and novel associations with other CNS disorders such as neurodegeneration, relapsing symptoms post-herpes simplex virus encephalitis, and demyelinating diseases. In addition, an algorithmic approach to detect and characterize neuronal cell surface autoantibodies is proposed.

## Introduction

Antibody-associated neuronal autoimmune disorders are a heterogenous group of syndromes that result from an autoimmune reaction to neuronal antigens. These disorders can broadly be divided into two main groups: classic paraneoplastic neurological syndromes (PNS) that associate with autoantibodies to intracellular neuronal antigens (e.g., Hu, Yo, Ri) and autoimmune encephalitis (AIE) that associate with autoantibodies to neuronal surface or synaptic antigens (1). The PNS typically occur in the context of cancer and almost always affect adults. These disorders result from an immunological response against tumor antigens that is misdirected to similar antigens expressed in the CNS. The autoantibodies against intracellular antigens have not been shown to be pathogenic. Patients with these autoantibodies show limited or no response to immunotherapy. In contrast, the AIE occur in association with autoantibodies against easily accessible antigens on the neuronal surface, such as synaptic receptors and membrane antigens [e.g., NMDAR, AMPAR, GABA(B)R] and the binding of the autoantibodies to their targets directly causes neuronal dysfunction. These diseases mainly affect young adults, adolescents, and children but can also occur in older individuals. The disorders may mimic psychiatric illness, dementia, or viral encephalitis, which often confuses and delays the diagnosis. Each of the identified autoantibodies associate with either a specific syndrome or a characteristic repertoire of symptoms and the detection of these autoantibodies confirms the diagnosis. This is important as patients with AIE often respond to immunotherapy. The following sections will give an overview of recent advances in AIE associated with neuronal cell surface autoantibodies with emphasis on pathogenesis, clinical presentation, and diagnostic approach.

## Pathogenetic Mechanisms

Most data on pathogenetic mechanisms associated with the neuronal cell surface autoantibodies focus on the NMDAR-antibodies found in patients with anti-NMDAR encephalitis. These autoantibodies recognize an extracellular, conformation-dependent epitope region close to aminoacid 369 of the GluN1 subunit of the NMDAR. The autoantibodies crosslink and internalize the NMDAR, which reduces the receptor density on the neuronal surface resulting in neuronal dysfunction (2). This process is reversible after removal of autoantibodies and may explain the good recovery of patients after immunotherapy (3). The NMDAR-antibodies that are highly specific for anti-NMDAR encephalitis should be distinguished from other types of glutamate receptor autoantibodies. For example, anti-double-stranded DNA (dsDNA) antibodies that cross-react with a linear epitope of the GluN2A and B subunits of the NMDAR have been found in 30–40% of patients with systemic lupus erythematodes (SLE) but whether these autoantibodies are responsible for neuropsychiatric symptoms of SLE remains a matter of controversy (4). Whereas the internalization of receptors was also described as an effect of autoantibodies found in AMPAR encephalitis (5), other AIE-associated autoantibodies may work through different mechanisms. For example, GABA(B)R-antibodies influence receptor function and block the inhibitory effects of baclofen on the spontaneous firing of cultured neurons, GABA(A)R-antibodies relocate the receptor from the synaptic to the extrasynaptic site, and LGI1-antibodies block the binding of LGI1 to ADAM22 that by unclear mechanisms results in a decrease of the AMPAR (1, 6). Future studies focusing on the antibody–receptor interaction will contribute to our understanding of immune mechanisms in AIE and could lead to more specific therapies in different stages of the disease.

## Clinical Presentation

Each of the currently known neuronal cell surface or synaptic autoantibodies associates with a specific syndrome or limited set of symptoms (Table 1). Anti-NMDAR encephalitis usually develops as a multistage process. Many patients have a prodromal syndrome and in a few days develop progressive anxiety, agitation, psychosis, memory deficits, and speech reduction. The disease then progresses to abnormal movements, coma, hypoventilation, and autonomic instability. In children, the behavioral change may be more difficult to detect, often with temper tantrums, hyperactivity, or irritability and there is a greater tendency for the first recognized symptom to be non-psychiatric, including seizures, abnormal movements, verbal reduction, or mutism (1, 3). The encephalitis associated with LGI1-, GABA(B)R-, and AMPAR-antibodies were originally described as classical limbic encephalitis but additional features might suggest the targeted antigen. For example, autoantibodies to LGI1 often associate with hyponatremia but rarely associate with a tumor, GABA(B)R-antibodies frequently occur with prominent seizures and about half of the patients have a small cell lung cancer (SCLC), while patients with AMPAR-antibodies often present with limbic encephalitis or psychiatric features (1, 5, 7). Some autoantibodies may associate with symptoms that extend beyond the CNS. For example, the encephalitis associated with DPPX-antibodies often starts with diarrhea or gastrointestinal dysfunction and substantial weight loss. This is then followed by the onset of neuropsychiatric and brainstem symptoms including symptoms of central nervous system hyperexcitability (seizures, tremulousness, myoclonus, nystagmus, hyperekplexia) (8) or a clinical picture resembling progressive encephalomyelitis with rigidity and myoclonus (PERM) (9).

**Table 1:** **Neuronal cell surface autoantibodies, associated tumors, and the clinical symptoms**.

Antigen	Tumor	Clinical symptoms
NMDAR	Ovarian teratoma (58% in patients >18 years)	Encephalitis
LGI1	Thymoma (<10%)	LE, myoclonia, hyponatremia
CASPR2	Thymoma (38%)	Encephalitis and/or Morvan syndrome
AMPAR	SCLC, breast, thymoma (60%)	LE, psychosis
GABA_B_R	SCLC (50%)	LE, ataxia
GABA_A_R	–	Status epilepticus, seizures, encephalitis
mGluR1	M. Hodgkin	Cerebellar ataxia
mGluR5	M. Hodgkin	Ophelia syndrome
DPPX (Kv4.1)	Follicular B cell lymphoma, CLL	Hallucinations, agitation, myoclonus, tremor, seizures, diarrhea
IgLON5	–	Non-REM and REM-sleep disorder, brainstem, and limbic dysfunction
GlyR	Lung cancer	SPS, PERM
Dopamine 2R	–	Basal ganglia encephalitis, Sydenham’s Chorea

*NMDAR, *N*-methyl-d-aspartate receptor; LGI1, leucine-rich glioma-inactivated 1; CASPR2, contactin-associated protein-like 2; AMPAR, amino-3-hydroxy-5-hydroxy-5-methyl-4-isoxazolepropionic acid receptor; GABA A/B R, gamma-aminobutyric acid A/B receptor; mGluR1/5, metabotropic glutamate receptor type 1/5; DPPX, dipeptidyl-peptidase-like protein-6; GlyR, Glycine receptor; CLL, chronic lymphatic leukemia; SCLC, small cell lung cancer; LE, limbic encephalitis; SPS, stiff-person syndrome; PERM, progressive encephalomyelitis with rigidity and myoclonus*.

An intriguing relation between autoimmunity and neurodegeneration has recently been discovered with the characterization of the IgLON5-antibody. These autoantibodies were detected in patients with prominent rapid-eye movement (REM) and non-REM-sleep dysfunction with abnormal behavior and movements, and brainstem symptoms with a chronic (or less frequently subacute) progressive disease course. Autopsy of two patients revealed deposits of tau that mainly affected neurons of the hypothalamus, thalamus, and brainstem. Although these findings suggest a link between IgLON5-antibodies and tau aggregation, it remains to be elucidated whether the autoantibodies are pathogenic or only an epiphenomenon (10). With the increased recognition of AIE novel associations with other disorders are emerging that connects different fields of medicine and emphasizes the importance of cross-disciplinary cooperation to ensure optimal management of the patients.

## Diagnostic Approach

### Available assays

Different techniques are available for the diagnosis of neuronal cell surface antibodies: tissue-based assays (TBA; in-house or commercially available), cell-based assay (CBA; in-house or commercially available), primary cultures of neurons (in-house), and immunoprecipitation (IP; in-house). In the TBA, rat or mouse brains are stained with CSF or serum of patients with an indirect immunhistochemistry or immunofluorescence technique. Anti-neuronal antibodies attach to their receptor or synaptic antigen in the rodent brain, resulting in a neuropil staining pattern in the hippocampus. In the CBA, cells (e.g., HEK293 cells) are transfected with the respective surface receptor or synaptic antigen and stained with CSF or serum of the patients with an indirect immunofluorescence technique. Autoantibodies against the specifically expressed receptor result in a membrane staining of the cells. Primary cultures of hippocampal neurons are stained with CSF or serum of patients with an indirect immunofluorescence technique and the autoantibodies are visualized as surface staining of neurons. In the IP, autoantibodies that are present in serum of patients bind to a specific antigen, the antigen–antibody complex is precipitated out of solution and measured.

**Figure 1:**
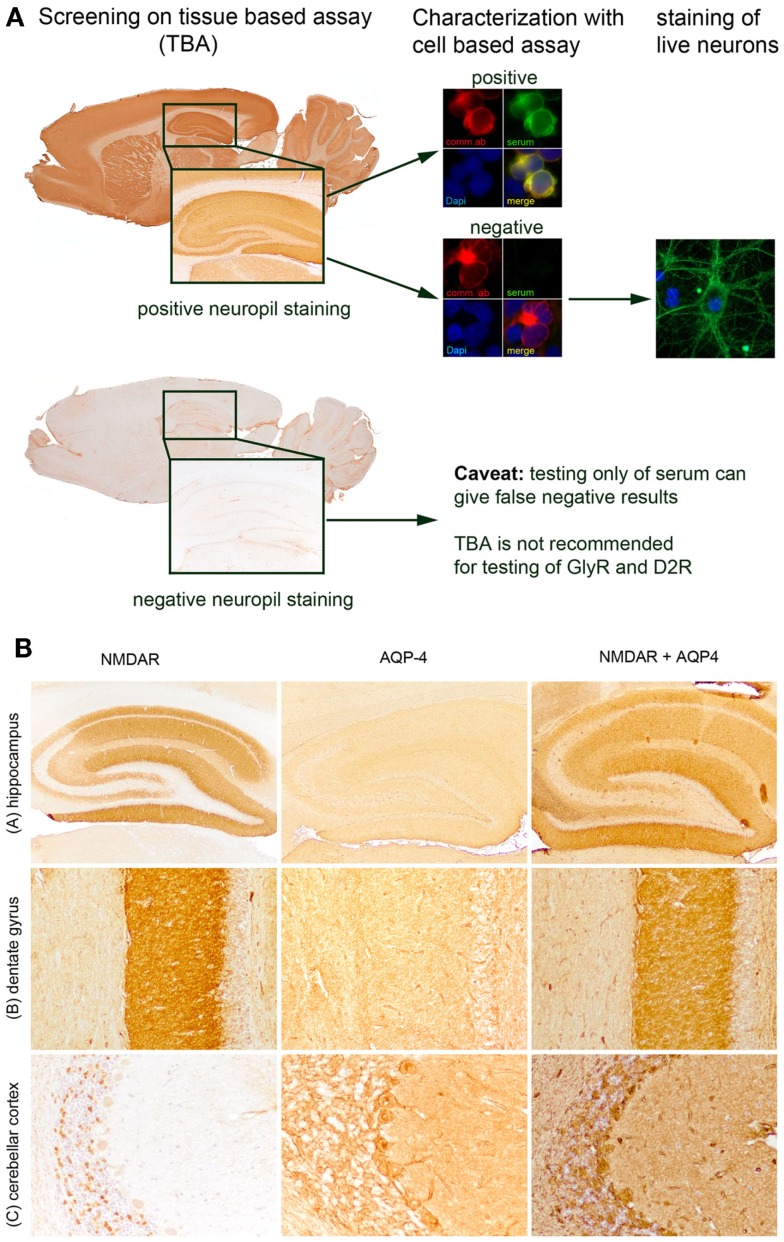
**Diagnosis of neuronal cell surface autoantibodies and overlapping syndromes**. Algorithmic approach for the diagnosis of neuronal cell surface autoantibodies **(A)** most of the currently known autoantibodies show an intensive neuropil staining in the hippocampus. The autoantibodies are subsequently characterized on HEK293 cells transfected with the antigen of interest (red: commercial antibody against the transfected antigen, green: patient’s serum with autoantibodies to the transfected antigen, blue: nuclear staining with DAPI). If all currently available cell-based assays remain negative (red: commercial antibody against the transfected antigen, green: patient’s serum without autoantibodies to the transfected antigen; blue: nuclear staining with DAPI) the sample may be stained on live hippocampal neurons. Glycin receptor- and D2R-antibodies may not be detectable by immunohistochemistry and should be tested directly with cell-based assays. Overlapping syndrome of anti-NMDAR encephalitis and neuromyelitis optica. **(B)** Identification of NMDAR-antibodies, aquaporin-4 (AQP-4), or both autoantibodies on tissue-based assay (TBA) showing the hippocampus **(A)**, the dentate gyrus of hippocampus [(B), enlarged from (A)], and cerebellar cortex (C). Examples of patients with autoantibodies targeting only the NMDAR (neuropil staining in hippocampus, dot-like staining in granular layer of cerebellar cortex), only the AQP-4 (mild neuropil staining in hippocampus, staining of glia limitans perivascularis, and reticular staining of granular layer of cerebellar cortex), and both, NMDAR and AQP-4 (strong neuropil staining in hippocampus, staining of glia limitans perivascularis, reticular staining of granular layer of cerebellar cortex). Magnification: (A): ×20, (B,C): ×100.

Most laboratories use the CBA for the diagnosis of neuronal cell surface autoantibodies, which is a highly sensitive and specific assay but bears the disadvantage that new autoantibodies are not detected. The TBA provides an excellent screening method, which detects most of the currently known neuronal cell surface autoantibodies (with some limitations for the GlyR- and D2R-antibodies) and can reveal new autoantibodies. To reach a maximum of sensitivity and specificity, a combination of TBA as screening method and CBA as confirmatory test may be considered (Figure 1A). Staining of hippocampal neurons and IP is mainly used in research but may be helpful in selected individual cases (e.g., in samples positive in TBA but negative in CBA to characterize and ascertain that the patient’s autoantibodies recognize a yet to be identified cell surface antigen).

### Detection of antibodies and interpretation of results

The sensitivity and specificity of serum and CSF testing has only been investigated in a few AIE, mainly anti-NMDAR encephalitis. While at the time of diagnosis of this disease autoantibodies are always present in CSF, the serum can be negative in up to 14% of patients, suggesting that serum examination alone may be insufficient to exclude AIE (2). On the other hand, the determination of autoantibodies only in serum carries the risk of diagnostic errors, due to false interpretations regardless of whether the CBA is performed with live or fixed cells. For example, a recent case report described a patient that for more than 1 year was considered to have anti-NMDAR encephalitis based on a positive test in serum (CSF was not tested). When studied for a second opinion, the clinical history and examination were not characteristic of anti-NMDAR encephalitis and her serum and CSF was negative for NMDAR-antibodies. She was eventually diagnosed with narcolepsy–cataplexy (11). Other problems with serum testing alone were demonstrated by a study using a live CBA showing that 23% of patients with serum antibodies did not have anti-NMDAR encephalitis (12). These problems do not occur with CSF analysis; when antibodies are identified in CSF, the patients have or have had anti-NMDAR encephalitis or a HSV-induced AIE (13). The experience with other autoantibodies to synaptic proteins is limited but seems to be similar; for example, all patients with encephalitis with GABA(A)R-antibodies in CSF show a similar syndrome characterized by prominent and refractory seizures and cortical–subcortical FLAIR MRI abnormalities; however, if antibodies are only found in serum, the variety of symptoms is more extensive, questioning if there is a real association (opsoclonus, stiff-person syndrome, among others). In contrast, LGI1-antibodies seem to be more prevalent in serum (although most patients have also CSF autoantibodies). Overall, these findings suggest that (1) physicians should know about the autoantibody-syndrome association, (2) not all autoantibodies have the same degree of sensitivity and specificity for AIE, (3) to minimize errors of interpretation and misleading diagnoses, all patients with encephalitis should have the serum and CSF tested not only for routine studies (proteins, glucose, cells, and PCR for exclusion of viruses) but also for autoantibodies.

Consecutive dilution steps of serum or CSF in TBA or CBA can be used to determine autoantibody titers (the endpoint where staining is still visible is indicated as the respective titer). The relation between autoantibody titers, relapses, and outcome was extensively investigated for anti-NMDAR encephalitis (2). For example, it has been shown that high autoantibody titers were associated with a poorer outcome or the presence of a teratoma. In contrast, a rapid decrease of CSF autoantibody titers within the first month of disease associated with a better prognosis. However, for the individual patient, autoantibody titers have limited value to guide treatment decisions, but may be helpful in instances such as determination of an active disease in patients with prolonged clinical course or assessment of relapses. For these purposes, CSF autoantibody titers were found to correlate better with the course of the disease than serum titers.

A recent study characterized CXCL13 as a potential biomarker for anti-NMDAR encephalitis. The CXCL13 is a cytokine produced by plasma cells and monocytes/macrophages. Seventy percent of patients with new onset of anti-NMDAR encephalitis had elevated CXCL13 levels in the CSF. Prolonged elevation of CSF CXCL13 (4–6 weeks) was an indicator of limited response to therapy suggesting that these patients could be candidates for more aggressive immunotherapy (14).

## Trigger for Autoimmune Encephalitis

The CNS is an immune-privileged organ, protected by the blood–brain barrier. Recognition of a specific antigen during immune surveillance results in the activation of inflammatory cells and release of inflammatory cytokines and chemokines that alter tissue barrier and initiate inflammation. Antigens such as neuronal membrane antigens do not activate the immune system under physiological conditions, but if activation is induced elsewhere, they can become targets for an autoimmune attack. A number of mechanisms were identified that could account for breaking immune tolerance in AIE (Table 1).

### Tumors as trigger for AIE

One possible trigger factor of AIE is the presence of a systemic tumor that expresses the neuronal antigen and studies have identified the expression of the targeted receptor subunit in the tumors of patients with anti-NMDAR, AMPAR, and GABA(B)R encephalitis (1, 5, 7). The hypothesis is that the systemic expression of these neuronal antigens initiates an immune response that is subsequently misdirected against the brain.

### Infection as trigger for AIE

Around 20% of patients with herpes simplex virus encephalitis (HSVE) have relapsing symptoms without evidence for viral reactivation (negative virus PCR in CSF, no new necrotic lesions in the MRI, and no response to anti-viral treatment). These episodes often manifest as choreic-like movements (chorea, orofacial dyskinesias, dystonia, or ballismus) accompanied by behavioral changes and were considered as immune-mediated. Recent studies found autoantibodies to the NMDAR, D2R, and other cell surface or synaptic proteins (antigens still unknown) in some of these patients, suggesting that HSVE may be a trigger for AIE (13). The precise mechanisms that initiate the immune response in anti-NMDAR encephalitis post-HSVE are not entirely clear. One possibility is molecular mimicry, in which autoantibodies generated in response to the virus cross-react with the neuronal antigen. Another explanation could be a viral-induced lysis of infected neurons with release of antigens in the presence of extensive inflammatory infiltrates. In this case, it would not be surprising that NMDAR-antibodies might be identified also in context with other viral infections.

### Other factors

In a considerable percentage of patients with AIE, the triggering factor remains unknown. Studies on different AIE established a marked predominance in females, suggesting a hormonal component of the disease although this may relate more to the known increased rate of autoimmunity in women. Genetic determinants likely contribute to susceptibility to autoimmunity. For example, specific alleles of HLA-DQB1 and HLA-DRB1 were preferentially found in patients with IgLON5-antibodies (10). Patients with AIE may have a propensity for autoimmune diseases as some have additional autoantibodies. Interestingly, anti-nuclear-, anti-dsDNA-, anti-cardiolipin-, or anti-TPO-antibodies are often found in serum of patients with AIE (5, 7). However, these concurrent autoantibodies are not linked to any particular neurological manifestation or syndrome and their significance remains to be established. A recent study of patients with anti-NMDAR encephalitis noted that some patients with AIE have inflammatory demyelinating CNS disease at times with aquaporin-4- or MOG-antibodies, occurring before, concurrent, or after the AIE diagnosis (Figure 1B) (15). The exact frequency of co-existing AIE and demyelinating disease is unknown as well as whether there is an underlying relationship between the two. Oligodendrocytes do express NMDAR but a role of NMDAR-antibodies in myelin dysfunction has not been studied.

## Conclusion

Recent neuroimmunological studies have provided fundamental insights into disease pathogenesis of AIE and explanations for the wide spectrum of syndromes. The mechanisms by which autoantibodies interfere with synaptic transmission are diverse but all share the potential reversibility of the dysfunction, explaining in part the good recovery of many patients after immunotherapy. These disorders result in different clinical phenotypes and their description has led to novel associations with other CNS diseases (e.g., psychosis, relapsing symptoms post-HSVE, demyelinating disease) making these patients an interdisciplinary challenge for treating physicians. Neuronal cell surface autoantibodies when tested appropriately are highly sensitive and specific diagnostic markers for AIE and have important implications for treatment decision and long-term patient management. Recognition of the specific AIE is important as these disorders may have different co-morbidities or associated tumors that need to be considered in the overall management of the patients.

## Conflict of Interest Statement

The author declares that the research was conducted in the absence of any commercial or financial relationships that could be construed as a potential conflict of interest.

## Abbreviations

AMPAR, amino-3-hydroxy-5-hydroxy-5-methyl-4-isoxazolepropionic acid receptor; ANA, anti-nuclear antibodies; CASPR2, contactin-associated protein-like 2; D2R, dopamine-2 receptor; DPPX, dipeptidyl-peptidase-like protein-6; dsDNA, double-stranded DNA; GABA A/B R, gamma-aminobutyric acid A/B receptor; GlyR, glycine receptor; LGI1, leucine-rich glioma-inactivated 1; mGluR1/5, metabotropic glutamate receptor type 1/5; NMDAR, *N*-methyl-d-aspartate receptor; TPO, thyroid peroxidase.
